# Supplementary material for: Genotypic and Phenotypic Structure of the Population of Phytophthora infestans in Egypt Revealed the Presence of European Genotypes
Source: J Fungi (Basel). 2022 Apr 30;8(5):468. doi: 10.3390/jof8050468 (PMC9147654; doi:10.3390/jof8050468)
Supplement: Supplementary file 1 [file jof-08-00468-s001.zip › jof-1707931-supplementary.pdf]

Supplementary data

Genotypic and phenotypic structure of the population of *Phytophthora infestans* in Egypt revealed the presence of European genotypes

Sherif Mohamed El-Ganainy[1,2*], Zafar Iqbal[3], Hossam Mohamed Awad[4], Muhammad Naeem Sattar[3], Abdel Mohsen Tohamy[2], Ahmed O. Abbas[5], Julie Squires[6] and David E L Cooke[6*]

[1]Department of Arid Land Agriculture, College of Agriculture and Food Sciences, King Faisal University, P.O. Box 420, Al-Ahsa 31982, Saudi Arabia; salganainy@kfu.edu.sa (S.M.E.-G.);

[2]Vegetable Diseases Research Department, Plant Pathology Research Institute, ARC, Giza 12619, Egypt; tohamy12@hotmail.com (A.M.T.)

[3]Central laboratories, King Faisal University, Saudi Arabia. mnsattar@kfu.edu.sa (M.N.S.);
zafar@kfu.edu.sa (Z.I.)

[4]Agriculture Botany Department, Menofia University, Shibin El-Kom, Egypt; Hosamawad80@yahoo.com (H.M.A.)

[5]Department of Animal and Fish Production, College of Agricultural and Food Sciences, King Faisal University, P.O. Box 420, Al-Ahsa 31982, Saudi Arabia; aabbas@kfu.edu.sa (A.O.A.)

[6]The James Hutton Institute, Dundee, Scotland, U.K; david.cooke@hutton.ac.uk (D.E.C.), julie.squires@hutton.ac.uk (J.S.)

*Correspondence: salganainy@kfu.edu.sa (S.M.E.-G.); david.cooke@hutton.ac.uk (D.E.C.)

Table S1. Differential set of *Solanum demissum* used in the study

Clone	Known R gene	Identity
Ma R1	R1	CEBECO 43154-5
Ma R2	R2	CEBECO 44158-4
Ma R3	R3a, R3b	CEBECO 4642-1
Ma R4	R4	CEBECO 4431-5
Ma R5	R5	Black 3053-18
Ma R6	R6	Black XD2-21
Ma R7	R7	Black 2182 ef (7)
Ma R8	R8	Black 2424 a (5)
Ma R9	R9 or R1R2R3R9	Black 2573 (2)
Ma R10	R10	Black 3681 ad (1)
Ma R11	R11	Black 5008 ab (6)

Table S2. Primer list for the 12-Plex assay used in the current study.

SSR locus	Dye	Product size range (bp)	Primer sequence (5′-3′)
G11	NED	115-210	Fwd-TGCTATTTATCAAGCGTGGG<br>Rev-GTTTCAATCTGCAGCCGTAAGA
PinfSSR3 (Pi02)	NED	255-275	Fwd-NED-ACTTGCAGAACTACCGCCC<br>Rev-GTTTGACCACTTTCCTCGGTTC
PinfSSR11	NED	325-360	Fwd -NED-TTAAGCCACGACATGAGCTG<br>Rev-GTTTAGACAATTGTTTTGTGGTCGC
D13	FAM	100-210	Fwd-FAM-TGCCCCCTGCTCACTC<br>Rev-GCTCGAATTCATTTTACAGACTTG
PinfSSR8	FAM	240-255	Fwd-FAM-AATCTGATCGCAACTGAGGG<br>Rev-GTTTACAAGATACACACGTCGCTCC
PinfSSR4	FAM	280-305	Fwd-FAM-TCTTGTTCGAGTATGCGACG<br>Rev-GTTTCACTTCGGGAGAAAGGCTTC
Pi04	VIC	155-175	Fwd-VIC-AGCGGCTTACCGATGG<br>Rev-GTTTCAGCGGCTGTTTCGAC
Pi70	VIC	185-205	Fwd-VIC-ATGAAAATACGTCAATGCTCG<br>Rev-CGTTGGATATTTCTATTTCTTCG
PinfSSR6	VIC	230-250	Fwd-GTTTTGGTGGGGCTGAAGTTTT<br>Rev-VIC - TCGCCACAAGATTTATTCCG
Pi63	VIC	265-280	FwdVIC – ATGACGAAGATGAAAGTGAGG<br>Rev-CGTATTTTCCTGTTTATCTAACACC
PinfSSR2	PET	165-180	FwdPET-CGACTTCTACATCAACCGGC<br>Rev-GTTTGCTTGGACTGCGTCTTTAGC
Pi4B	PET	200-295	FwdPET – AAAATAAAGCCTTTGGTTCA<br>Rev-GCAAGCGAGGTTTGTAGATT

Table S3. Euroblight Sample sheet

Country ID	User Init	Surveyor Name	Institution Name	Reference Number/Name	Host Genus ID	Obs Year	Month	Day	Location Name	Latitude	Longitude	Survey Site ID	Host Cultivar Name	Growth Stage ID	Severity
EG	SGA	Sherif El-Ganainy (SEG)	Plant Pathology Research Institute	EG-001	1	2010	1	5	Nobareyah	30.6436	30.1510	1	Hermes	10	4
EG	SGA	SEG	PPRI	EG-002	1	2010	1	7	Tamalay	30.5112	30.8367	1	Spunta	31	6
EG	SGA	SEG	PPRI	EG-003	1	2010	1	7	Minuf	30.4677	30.9241	1	Agria	39	11
EG	SGA	SEG	PPRI	EG-004	1	2010	1	7	Minuf	30.4829	30.9192	1	Lady Rosetta	39	9
EG	SGA	SEG	PPRI	EG-005	1	2010	1	12	Tamalay	30.5015	30.8596	1	Diamant	10	11
EG	SGA	SEG	PPRI	EG-006	1	2010	1	20	Al Qanater	30.1867	31.1313	1	Nicola	39	2
EG	SGA	SEG	PPRI	EG-007	1	2010	1	25	Al-Busayli	31.3289	30.4069	1	Spunta	31	7
EG	SGA	SEG	PPRI	EG-008	1	2010	1	27	Minuf	30.4511	30.9285	1	Bellini	39	5
EG	SGA	SEG	PPRI	EG-009	1	2010	2	1	Al Tawfikeya	30.6090	30.7677	1	Slaney	33	4
EG	SGA	SEG	PPRI	EG-010	1	2010	2	3	Minuf	30.4435	30.9257	1	Lady Rosetta	39	6
EG	SGA	SEG	PPRI	EG-011	1	2010	2	5	Tamalay	30.4967	30.8560	1	Spunta	33	5
EG	SGA	SEG	PPRI	EG-012	1	2010	2	12	El-Mahalla El-Kubra	30.9388	31.1202	1	Cara	65	5
EG	SGA	SEG	PPRI	EG-013	1	2010	2	26	Minuf	30.4667	30.9443	1	Burren	67	7
EG	SGA	SEG	PPRI	EG-014	1	2010	3	5	Al-Busayli	31.3277	30.4025	1	Slaney	39	10
EG	SGA	SEG	PPRI	EG-015	1	2010	3	23	Shirbin	31.2134	31.5308	1	Sante	65	9
EG	SGA	SEG	PPRI	EG-016	2	2010	4	15	Qelyoub	30.1639	31.2030	1	Super Strain B	67	6
EG	SGA	SEG	PPRI	EG-017	1	2010	12	10	kom hamada	30.7192	30.7324	1	Bellini	31	3
EG	SGA	SEG	PPRI	EG-018	1	2010	12	17	Elbostan	30.7951	30.4798	1	Inova	33	2
EG	SGA	SEG	PPRI	EG-019	1	2011	1	3	Tamalay	30.4941	30.8449	1	Diamant	15	11
EG	SGA	SEG	PPRI	EG-020	1	2011	1	4	Minuf	30.4631	30.9459	1	Agria	67	11
EG	SGA	SEG	PPRI	EG-021	1	2011	1	4	Minuf	30.4632	30.9491	1	Lady Rosetta	69	11
EG	SGA	SEG	PPRI	EG-022	1	2011	1	7	Tamalay	30.4965	30.8480	1	Spunta	63	9
EG	SGA	SEG	PPRI	EG-023	1	2011	1	7	Tamalay	30.4978	30.8459	1	Diamant	65	4

EG	SGA	SEG	PPRI	EG-024	1	2011	1	12	El-Mahalla El-Kubra	31.0006	31.1522	1	Cara	25	4
EG	SGA	SEG	PPRI	EG-025	1	2011	1	19	Minuf	30.4479	30.9297	1	Lady Rosetta	63	11
EG	SGA	SEG	PPRI	EG-026	1	2011	1	19	Minuf	30.4292	30.9335	1	Provento	51	4
EG	SGA	SEG	PPRI	EG-027	1	2011	1	19	Minuf	30.4510	30.9249	1	Agria	69	11
EG	SGA	SEG	PPRI	EG-028	1	2011	1	20	Tamalay	30.5199	30.8637	1	Diamant	15	9
EG	SGA	SEG	PPRI	EG-031	1	2011	1	22	Barheem	30.4561	30.8901	1	Diamant	69	10
EG	SGA	SEG	PPRI	EG-032	1	2011	1	22	Teta	30.5114	30.9129	1	Lady Rosetta	69	11
EG	SGA	SEG	PPRI	EG-034	1	2011	1	29	kom hamada	30.7592	30.6822	1	Accent	21	2
EG	SGA	SEG	PPRI	EG-036	1	2011	2	10	Minuf	30.4750	30.9330	1	Lady Rosetta	67	9
EG	SGA	SEG	PPRI	EG-037	1	2011	2	11	Tamalay	30.5110	30.8392	1	Agria	69	11
EG	SGA	SEG	PPRI	EG-038	1	2011	2	11	Tamalay	30.5107	30.8395	1	Spunta	25	2
EG	SGA	SEG	PPRI	EG-039	1	2011	2	11	Tamalay	30.5109	30.8422	1	Spunta	25	3
EG	SGA	SEG	PPRI	EG-040	1	2011	2	15	Minuf	30.4740	30.9334	1	Diamant	25	2
EG	SGA	SEG	PPRI	EG-041	1	2011	2	16	Tamalay	30.4924	30.8453	1	Spunta	25	6
EG	SGA	SEG	PPRI	EG-043	1	2011	2	16	Tamalay	30.4950	30.8446	1	Spunta	25	4
EG	SGA	SEG	PPRI	EG-044	1	2011	2	16	Tamalay	30.4967	30.8458	1	Spunta	25	4
EG	SGA	SEG	PPRI	EG-045	1	2011	2	16	Tamalay	30.4894	30.8679	1	Spunta	25	5
EG	SGA	SEG	PPRI	EG-046	1	2011	2	16	Minuf	30.4727	30.4727	1	Lady Rosetta	25	2
EG	SGA	SEG	PPRI	EG-047	1	2011	2	16	Tamalay	30.4990	30.8603	1	Diamant	67	9
EG	SGA	SEG	PPRI	EG-049	1	2011	2	16	Tamalay	30.5170	30.8418	1	Hermes	15	2
EG	SGA	SEG	PPRI	EG-050	1	2011	2	16	Menouf	30.4823	30.9059	1	Diamant	21	2
EG	SGA	SEG	PPRI	EG-051	1	2011	2	21	Nader	30.5284	30.8498	1	Spunta	25	3
EG	SGA	SEG	PPRI	EG-052	1	2011	2	23	Menouf	30.4574	30.9181	1	Lady Rosetta	21	2
EG	SGA	SEG	PPRI	EG-054	1	2011	2	23	Gazi	30.4578	30.8549	1	Spunta	21	2
EG	SGA	SEG	PPRI	EG-055	1	2011	2	23	Minuf	30.4592	30.9448	1	Lady Rosetta	30	4
EG	SGA	SEG	PPRI	EG-056	1	2011	2	23	Minuf	30.4588	30.9464	1	Lady Rosetta	21	4
EG	SGA	SEG	PPRI	EG-057	1	2011	2	23	Minuf	30.4595	30.9502	1	Lady Rosetta	21	4

EG	SGA	SEG	PPRI	EG-058	1	2011	2	23	Minuf	30.4591	30.9515	1	Lady Rosetta	30	4
EG	SGA	SEG	PPRI	EG-059	1	2011	2	26	Alqam	30.5410	30.8264	1	Agria	35	5
EG	SGA	SEG	PPRI	EG-060	1	2011	2	27	Minuf	30.4885	30.9101	1	Diamant	21	2
EG	SGA	SEG	PPRI	EG-061	1	2011	2	27	Minuf	30.4822	30.9295	1	Diamant	21	2
EG	SGA	SEG	PPRI	EG-062	1	2011	2	27	Minuf	30.4824	30.9325	1	Diamant	21	2
EG	SGA	SEG	PPRI	EG-063	1	2011	2	27	Minuf	30.4814	30.9346	1	Diamant	21	2
EG	SGA	SEG	PPRI	EG-064	1	2011	2	27	Minuf	30.4815	30.9362	1	Diamant	21	2
EG	SGA	SEG	PPRI	EG-065	1	2011	2	27	Minuf	30.4799	30.9380	1	Diamant	21	2
EG	SGA	SEG	PPRI	EG-067	1	2011	3	5	Nader	30.5260	30.8519	1	Spunta	25	2
EG	SGA	SEG	PPRI	EG-068	1	2011	3	6	Minuf	30.4397	30.9219	1	Lady Rosetta	30	2
EG	SGA	SEG	PPRI	EG-069	1	2011	3	6	Minuf	30.4379	30.9219	1	Lady Rosetta	30	2
EG	SGA	SEG	PPRI	EG-070	1	2011	3	6	Fisha	30.3999	30.9430	1	Draga	39	2
EG	SGA	SEG	PPRI	EG-072	1	2011	3	15	Minuf	30.4726	30.9454	1	Lady Rosetta	59	8
EG	SGA	SEG	PPRI	EG-073	1	2011	3	15	Minuf	30.4731	30.9474	1	Agria	67	11
EG	SGA	SEG	PPRI	EG-074	1	2011	3	16	Kamshoush	30.4174	30.9362	1	Spunta	59	7
EG	SGA	SEG	PPRI	EG-075	1	2011	3	16	El-Hamoul	30.4811	30.9674	1	Diamant	59	7
EG	SGA	SEG	PPRI	EG-076	1	2011	3	25	El-Bostan	30.8010	30.4771	1	Inova	39	3
EG	SGA	SEG	PPRI	EG-077	1	2011	3	25	El-Bostan	30.7882	30.4887	1	Princess	39	3
EG	SGA	SEG	PPRI	EG-078	1	2011	3	30	Minuf	30.4432	30.9312	1	Agria	67	11
EG	SGA	SEG	PPRI	EG-079	1	2011	4	5	Minuf	30.4491	30.9316	1	Diamant	63	8
EG	SGA	SEG	PPRI	EG-080	1	2011	4	5	El-Hamoul	30.4822	30.9610	1	Diamant	59	8
EG	SGA	SEG	PPRI	EG-081	1	2011	4	6	Kamshoush	30.4260	30.9349	1	Mondial	59	3
EG	SGA	SEG	PPRI	EG-082	1	2011	4	10	Tamalay	30.5204	30.8513	1	Burren	63	2
EG	SGA	SEG	PPRI	EG-083	1	2011	4	15	Shanshour	30.3615	30.9970	1	Spunta	59	7
EG	SGA	SEG	PPRI	EG-084	1	2011	4	25	Tamalay	30.4819	30.8377	1	Spunta	59	7
EG	SGA	SEG	PPRI	EG-085	1	2011	4	25	Tamalay	30.4841	30.8372	1	Agria	69	11
EG	SGA	SEG	PPRI	EG-086	1	2011	4	25	Tamalay	30.4925	30.8343	1	Diamant	59	6
EG	SGA	SEG	PPRI	EG-087	1	2011	4	26	Minuf	30.4459	30.9214	1	Lady Rosetta	63	8
EG	SGA	SEG	PPRI	EG-088	1	2011	4	28	Tamalay	30.4997	30.8326	1	Spunta	59	10
EG	SGA	SEG	PPRI	EG-089	2	2012	3	25	Sakha	31.0929	30.9503	1	GS12	59	5
EG	SGA	SEG	PPRI	EG-090	1	2012	4	8	El-Mahalla El-Kubra	30.9891	31.1214	1	Cara	67	6

EG	SGA	SEG	PPRI	EG-091	1	2012	4	20	Sakha	31.0944	30.9546	1	Spunta	67	10
EG	SGA	SEG	PPRI	EG-092	1	2012	5	2	El-Mahalla El-Kubra	31.0116	31.1145	1	Cara	67	5
EG	SGA	SEG	PPRI	EG-093	1	2012	5	2	El-Mahalla El-Kubra	31.0143	31.1025	1	Cara	67	5
EG	SGA	SEG	PPRI	EG-094	1	2012	5	2	El-Mahalla El-Kubra	30.9356	31.1094	1	Cara	67	5
EG	SGA	SEG	PPRI	EG-095	2	2012	5	10	Sakha	31.0836	30.9424	1	GS12	63	10
EG	SGA	SEG	PPRI	EG-096	1	2012	5	16	El-Mahalla El-Kubra	30.9215	31.0857	1	Cara	67	6

Table S4. SSR table

sample name	Isolate name	Country	Genotype	Allele 1 Pi02	Allele 2 Pi02	Allele 3 Pi02	Allele 1 Pi4B	Allele 2 Pi4B	Allele 3 Pi4B	Allele 1 G11	Allele 2 G11	Allele 3 G11	Allele 1 Pi04	Allele 2 Pi04	Allele 1 Pi63	Allele 2 Pi63	Allele 3 Pi63	Allele 1 Pi70	Allele 2 Pi70	Allele 1 D13	Allele 2 D13	Allele 3 D13	Allele 1 SSR11	Allele 2 SSR11	Allele 1 SSR2	Allele 2 SSR2	Allele 1 SSR4	Allele 2 SSR4	Allele 3 SSR4	Allele 1 SSR6	Allele 2 SSR6	Allele 3 SSR6	Allele 1 SSR8	Allele 2 SSR8	Allele 3 SSR8
09_7110A	C3	UK	13_A2_1	266	268	0	205	213	0	154	160	0	166	170	273	279	0	192	192	136	154	0	341	341	173	173	284	294	0	240	244	0	260	266	0
09_7126A	C_4	UK	6_A1	258	266	268	213	217	0	160	160	0	166	170	273	279	0	192	195	null	null	0	341	355	173	175	284	292	296	244	244	0	260	266	0
09_7186A	C_5	UK	13_A2_2	266	268	0	205	213	0	154	160	0	166	170	273	279	0	192	192	136	140	154	341	341	173	173	284	294	0	240	244	0	260	266	0
09_7262A	C_6	UK	8_2_A1	268	268	0	205	217	0	166	166	0	166	170	273	279	0	192	192	118	136	0	331	341	173	175	288	294	0	242	244	0	260	266	0
09_7410A	C_7	UK	13_A2_5	266	268	0	205	213	0	154	160	164	166	170	273	279	0	192	192	136	154	0	341	341	173	173	284	294	0	240	244	0	260	266	0
09_7610A	C_8	UK	13_A2_23	266	268	0	205	213	0	154	160	0	166	170	273	279	0	192	192	136	150	0	341	341	173	173	284	294	0	240	244	0	260	266	0
09_7710A	C_9	UK	23_A1_3	266	268	270	213	217	0	142	156	206	170	170	270	279	0	192	192	136	210	0	331	341	173	175	288	294	296	220	220	0	260	266	0
09_7722B	C_10	UK	23_A1_4	266	268	270	213	217	0	142	156	206	170	170	270	279	0	192	192	136	210	0	331	341	173	175	288	294	0	244	244	0	260	266	0
09_7770B	C_11	UK	10_A2	268	268	0	213	217	0	162	208	0	166	170	273	279	0	192	192	136	136	0	331	341	173	175	284	288	0	240	242	244	260	266	0
10_7814A	C_12	UK	23_A1_3	266	268	270	213	217	0	142	156	206	170	170	270	279	0	192	192	136	210	0	331	341	173	175	288	294	296	244	244	0	260	266	0
10_7930C	C_13	UK	13_A2_15	266	268	0	205	213	0	154	160	0	166	170	273	279	0	192	192	136	156	0	341	341	173	173	284	294	0	240	244	0	260	266	0
10_8058A	C_14	UK	2_A1	258	268	0	217	217	0	154	156	206	166	170	270	273	279	192	195	132	132	0	341	355	173	173	288	290	292	242	244	0	260	264	266
C4	C_15	UK	5_A1	268	268	0	205	217	0	156	162	0	166	170	279	279	0	192	192	136	136	0	341	355	175	175	292	294	0	244	244	0	266	266	0

EG_10_01_Nub1.1	EG_01	Egypt	13_A2_5	266	268	0	205	213	0	154	160	164	166	170	273	279	0	192	192	136	154	0	341	341	173	173	284	294	0	240	244	0	260	266	0
EG_10_02_Tam1.1	EG_02	Egypt	13_A2_5	266	268	0	205	213	0	154	160	164	166	170	273	279	0	192	192	136	154	0	341	341	173	173	284	294	0	240	244	0	260	266	0
EG_10_03_Men1.1	EG_03	Egypt	23_A1_19	266	270	0	213	213	0	142	206	0	170	170	279	279	0	192	192	136	210	0	331	341	173	173	288	294	0	244	244	0	260	266	0
EG_10_04_MEN2.1	EG_04	Egypt	23_A1_15	266	268	270	213	217	0	156	210	0	170	170	270	279	0	192	192	136	214	0	331	341	173	175	288	294	0	244	244	0	260	266	0
EG_10_05_Tam2.1S.F	EG_05	Egypt	13_A2_5	266	268	0	205	213	0	154	160	164	166	170	273	279	0	192	192	136	154	0	341	341	173	173	284	294	0	240	244	0	260	266	0
EG_10_06_Elk1.1	EG_06	Egypt	misc	268	268	0	205	217	0	160	204	0	166	170	270	273	279	192	192	118	136	0	341	341	173	175	284	294	0	240	242	244	260	260	0
EG_10_07_ELS1.1	EG_07	Egypt	23_A1_4	266	268	270	213	217	0	142	156	206	170	170	270	279	0	192	192	136	210	0	331	341	173	175	288	294	0	244	244	0	260	266	0
EG_10_08_Men3.1	EG_08	Egypt	23_A1_15	266	268	270	213	217	0	156	210	0	170	170	270	279	0	192	192	136	214	0	331	341	173	175	288	294	0	244	244	0	260	266	0
EG_10_09_Taw1.1	EG_09	Egypt	23_A1_15	266	268	270	213	217	0	156	210	0	170	170	270	279	0	192	192	136	214	0	331	341	173	175	288	294	0	244	244	0	260	266	0
EG_10_10_Men4.1	EG_10	Egypt	23_A1_15	266	268	270	213	217	0	156	210	0	170	170	270	279	0	192	192	136	214	0	331	341	173	175	288	294	0	244	244	0	260	266	0
EG_10_11_Tam3.1	EG_11	Egypt	23_A1_15	266	268	270	213	217	0	156	210	0	170	170	270	279	0	192	192	136	214	0	331	341	173	175	288	294	0	244	244	0	260	266	0
EG_10_12_Mah1.1	EG_12	Egypt	23_A1_16	266	268	270	213	213	0	156	210	0	170	170	270	279	0	192	192	140	214	0	331	341	173	173	288	294	0	244	244	0	260	266	0
EG_10_13_Men5.1	EG_13	Egypt	23_A1_15	266	268	270	213	217	0	156	210	0	170	170	270	279	0	192	192	136	214	0	331	341	173	175	288	294	0	244	244	0	260	266	0
EG_10_14_Els2.1	EG_14	Egypt	13_A2_5	266	268	0	205	213	0	154	160	164	166	170	273	279	0	192	192	136	154	0	341	341	173	173	284	294	0	240	244	0	260	266	0
EG_10_15_She1.1	EG_15	Egypt	13_A2_43	266	268	0	205	213	0	154	160	164	166	170	273	279	0	192	192	136	156	0	341	341	173	173	284	294	0	240	244	0	260	266	0
EG_10_16_KAL1.1-Tomato	EG_16	Egypt	23_A1_15	266	268	270	213	217	0	156	210	0	170	170	270	279	0	192	192	136	214	0	331	341	173	175	288	294	0	244	244	0	260	266	0
EG_11_17_kom1.1	EG_17	Egypt	13_A2_84	266	272	0	205	213	0	154	156	160	166	170	273	279	0	192	192	134	136	154	341	341	173	173	284	294	0	240	244	0	260	266	0
EG_11_18_Elb2.1	EG_18	Egypt	13_A2_84	266	272	0	205	213	0	154	156	160	166	170	273	279	0	192	192	134	136	154	341	341	173	173	284	294	0	240	244	0	260	266	0
EG_11_19_Tam4.1	EG_19	Egypt	23_A1_15	266	268	270	213	217	0	156	210	0	170	170	270	279	0	192	192	136	214	0	331	341	173	175	288	294	0	244	244	0	260	266	0
EG_11_20_Men6.1	EG_20	Egypt	23_A1_15	266	268	270	213	217	0	156	210	0	170	170	270	279	0	192	192	136	214	0	331	341	173	175	288	294	0	244	244	0	260	266	0
EG_11_21_Men7.1S.F	EG_21	Egypt	13_A2_5	266	268	0	205	213	0	154	160	164	166	170	273	279	0	192	192	136	154	0	341	341	173	173	284	294	0	240	244	0	260	266	0
EG_11_22_Tam5.1	EG_22	Egypt	23_A1_15	266	268	270	213	217	0	156	210	0	170	170	270	279	0	192	192	136	214	0	331	341	173	175	288	294	0	244	244	0	260	266	0
EG_11_23_Tam6.1fta	EG_23	Egypt	23_A1_15	266	268	270	213	217	0	156	210	0	170	170	270	279	0	192	192	136	214	0	331	341	173	175	288	294	0	244	244	0	260	266	0
EG_11_24_Mah2.1	EG_24	Egypt	13_A2_84	266	272	0	205	213	0	154	156	160	166	170	273	279	0	192	192	134	136	154	341	341	173	173	284	294	0	240	244	0	260	266	0
EG_11_25_Men8.1	EG_25	Egypt	23_A1_10	266	268	270	213	217	0	142	156	208	170	170	270	279	0	192	192	136	210	0	331	341	173	175	288	294	0	244	244	0	260	266	0
EG_11_26_Men9.1	EG_26	Egypt	13_A2_84	266	272	0	205	213	0	154	156	160	166	170	273	279	0	192	192	134	136	154	341	341	173	173	284	294	0	240	244	0	260	266	0
EG_11_27_Men10.1	EG_27	Egypt	23_A1_15	266	268	270	213	217	0	156	210	0	170	170	270	279	0	192	192	136	214	0	331	341	173	175	288	294	0	244	244	0	260	266	0
EG_11_28_Tam7.1	EG_28	Egypt	23_A1_15	266	268	270	213	217	0	156	210	0	170	170	270	279	0	192	192	136	214	0	331	341	173	175	288	294	0	244	244	0	260	266	0

EG_11_31_Bar1.1	EG_31	Egypt	23_A1_13	266	268	270	213	217	0	142	156	210	170	170	270	279	0	192	192	136	136	0	331	341	173	175	288	294	0	244	244	0	260	266	0
EG_11_32_Tet1.1	EG_32	Egypt	23_A1_14	266	268	270	213	217	0	142	156	210	170	170	270	279	0	192	192	142	210	0	331	341	173	175	288	294	0	244	244	0	260	266	0
EG_11_34_Kom2.1	EG_34	Egypt	23_A1_15	266	268	270	213	217	0	156	210	0	170	170	270	279	0	192	192	136	214	0	331	341	173	175	288	294	0	244	244	0	260	266	0
EG_11_36_Men11.1	EG_36	Egypt	23_A1_15	266	268	270	213	217	0	156	210	0	170	170	270	279	0	192	192	136	214	0	331	341	173	175	288	294	0	244	244	0	260	266	0
EG_11_37_Tam11.1	EG_37	Egypt	23_A1_15	266	268	270	213	217	0	156	210	0	170	170	270	279	0	192	192	136	214	0	331	341	173	175	288	294	0	244	244	0	260	266	0
EG_11_38_Tam12.1	EG_38	Egypt	23_A1_15	266	268	270	213	217	0	156	210	0	170	170	270	279	0	192	192	136	214	0	331	341	173	175	288	294	0	244	244	0	260	266	0
EG_11_39_TAM13.1	EG_39	Egypt	23_A1_15	266	268	270	213	217	0	156	210	0	170	170	270	279	0	192	192	136	214	0	331	341	173	175	288	294	0	244	244	0	260	266	0
EG_11_40_Men12.1	EG_40	Egypt	23_A1_17	268	270	0	213	217	0	156	210	0	170	170	270	279	0	192	192	136	214	0	331	341	173	175	288	294	0	244	244	0	260	266	0
EG_11_41_Tam14.1	EG_41	Egypt	23_A1_15	266	268	270	213	217	0	156	210	0	170	170	270	279	0	192	192	136	214	0	331	341	173	175	288	294	0	244	244	0	260	266	0
EG_11_43_Tam16.1	EG_43	Egypt	23_A1_15	266	268	270	213	217	0	156	210	0	170	170	270	279	0	192	192	136	214	0	331	341	173	175	288	294	0	244	244	0	260	266	0
EG_11_44_Tam17.1	EG_44	Egypt	23_A1_15	266	268	270	213	217	0	156	210	0	170	170	270	279	0	192	192	136	214	0	331	341	173	175	288	294	0	244	244	0	260	266	0
EG_11_45_Tam18.1	EG_45	Egypt	23_A1_15	266	268	270	213	217	0	156	210	0	170	170	270	279	0	192	192	136	214	0	331	341	173	175	288	294	0	244	244	0	260	266	0
EG_11_46_Men13.1	EG_46	Egypt	23_A1_15	266	268	270	213	217	0	156	210	0	170	170	270	279	0	192	192	136	214	0	331	341	173	175	288	294	0	244	244	0	260	266	0
EG_11_47_Tam19.1	EG_47	Egypt	23_A1_15	266	268	270	213	217	0	156	210	0	170	170	270	279	0	192	192	136	214	0	331	341	173	175	288	294	0	244	244	0	260	266	0
EG_11_49_Tam20.1	EG_49	Egypt	23_A1_15	266	268	270	213	217	0	156	210	0	170	170	270	279	0	192	192	136	214	0	331	341	173	175	288	294	0	244	244	0	260	266	0
EG_11_50_Men15.1	EG_50	Egypt	23_A1_15	266	268	270	213	217	0	156	210	0	170	170	270	279	0	192	192	136	214	0	331	341	173	175	288	294	0	244	244	0	260	266	0
EG_11_51_Nad1.1	EG_51	Egypt	23_A1_12	266	268	270	213	217	0	142	156	210	170	170	270	279	0	192	192	136	210	0	331	341	173	175	288	294	0	244	244	0	260	266	0
EG_11_52_Men16.1	EG_52	Egypt	23_A1_15	266	268	270	213	217	0	156	210	0	170	170	270	279	0	192	192	136	214	0	331	341	173	175	288	294	0	244	244	0	260	266	0
EG_11_54_Gez1.1	EG_54	Egypt	23_A1_15	266	268	270	213	217	0	156	210	0	170	170	270	279	0	192	192	136	214	0	331	341	173	175	288	294	0	244	244	0	260	266	0
EG_11_55_Men18.1	EG_55	Egypt	23_A1_15	266	268	270	213	217	0	156	210	0	170	170	270	279	0	192	192	136	214	0	331	341	173	175	288	294	0	244	244	0	260	266	0
EG_11_56_Men19.1S.F	EG_56	Egypt	13_A2_84	266	272	0	205	213	0	154	156	160	166	170	273	279	0	192	192	134	136	154	341	341	173	173	284	294	0	240	244	0	260	266	0
EG_11_57_MEN20.1	EG_57	Egypt	23_A1_15	266	268	270	213	217	0	156	210	0	170	170	270	279	0	192	192	136	214	0	331	341	173	175	288	294	0	244	244	0	260	266	0
EG_11_58_Men21.1	EG_58	Egypt	23_A1_15	266	268	270	213	217	0	156	210	0	170	170	270	279	0	192	192	136	214	0	331	341	173	175	288	294	0	244	244	0	260	266	0
EG_11_60_Men22.1	EG_60	Egypt	23_A1_15	266	268	270	213	217	0	156	210	0	170	170	270	279	0	192	192	136	214	0	331	341	173	175	288	294	0	244	244	0	260	266	0
EG_11_61_Men23.1S.F	EG_61	Egypt	13_A2_84	266	272	0	205	213	0	154	156	160	166	170	273	279	0	192	192	134	136	154	341	341	173	173	284	294	0	240	244	0	260	266	0
EG_11_62_Men23.2	EG_62	Egypt	13_A2_84	266	272	0	205	213	0	154	156	160	166	170	273	279	0	192	192	134	136	154	341	341	173	173	284	294	0	240	244	0	260	266	0
EG_11_63_Men24.1	EG_63	Egypt	23_A1_15	266	268	270	213	217	0	156	210	0	170	170	270	279	0	192	192	136	214	0	331	341	173	175	288	294	0	244	244	0	260	266	0
EG_11_64_Men25.1	EG_64	Egypt	13_A2_84	266	272	0	205	213	0	154	156	160	166	170	273	279	0	192	192	134	136	154	341	341	173	173	284	294	0	240	244	0	260	266	0

EG_11_65_Men26.1	EG_65	Egypt	23_A1_12	266	268	270	213	217	0	142	156	210	170	170	270	279	0	192	192	136	210	0	331	341	173	175	288	294	0	244	244	0	260	266	0
EG_11_67_Nad2.1	EG_67	Egypt	23_A1_15	266	268	270	213	217	0	156	210	0	170	170	270	279	0	192	192	136	214	0	331	341	173	175	288	294	0	244	244	0	260	266	0
EG_11_68_MEN27.1sf	EG_68	Egypt	13_A2_84	266	272	0	205	213	0	154	156	160	166	170	273	279	0	192	192	134	136	154	341	341	173	173	284	294	0	240	244	0	260	266	0
EG_11_69_Men28.1	EG_69	Egypt	23_A1_15	266	268	270	213	217	0	156	210	0	170	170	270	279	0	192	192	136	214	0	331	341	173	175	288	294	0	244	244	0	260	266	0
EG_11_70_Fes1.1	EG_70	Egypt	13_A2_84	266	272	0	205	213	0	154	156	160	166	170	273	279	0	192	192	134	136	154	341	341	173	173	284	294	0	240	244	0	260	266	0
EG_11_72_Men29.1	EG_72	Egypt	23_A1_12	266	268	270	213	217	0	142	156	210	170	170	270	279	0	192	192	136	210	0	331	341	173	175	288	294	0	244	244	0	260	266	0
EG_11_73_Men30.1	EG_73	Egypt	23_A1_15	266	268	270	213	217	0	156	210	0	170	170	270	279	0	192	192	136	214	0	331	341	173	175	288	294	0	244	244	0	260	266	0
EG_11_74_Kam1.1	EG_74	Egypt	23_A1_15	266	268	270	213	217	0	156	210	0	170	170	270	279	0	192	192	136	214	0	331	341	173	175	288	294	0	244	244	0	260	266	0
EG_11_75_Ham1.1.	EG_75	Egypt	23_A1_18	266	270	0	213	217	0	156	210	0	170	170	270	279	0	192	192	136	214	0	331	341	173	175	288	294	0	244	244	0	260	266	0
EG_11_76_Bos1.1S.F	EG_76	Egypt	13_A2_84	266	272	0	205	213	0	154	156	160	166	170	273	279	0	192	192	134	136	154	341	341	173	173	284	294	0	240	244	0	260	266	0
EG_11_77_Bos1.2	EG_77	Egypt	13_A2_84	266	272	0	205	213	0	154	156	160	166	170	273	279	0	192	192	134	136	154	341	341	173	173	284	294	0	240	244	0	260	266	0
EG_11_78_Men31.1	EG_78	Egypt	23_A1_15	266	268	270	213	217	0	156	210	0	170	170	270	279	0	192	192	136	214	0	331	341	173	175	288	294	0	244	244	0	260	266	0
EG_11_79_Men31.2	EG_79	Egypt	23_A1_15	266	268	270	213	217	0	156	210	0	170	170	270	279	0	192	192	136	214	0	331	341	173	175	288	294	0	244	244	0	260	266	0
EG_11_80_Ham1.2	EG_80	Egypt	23_A1_15	266	268	270	213	217	0	156	210	0	170	170	270	279	0	192	192	136	214	0	331	341	173	175	288	294	0	244	244	0	260	266	0
EG_11_81_Kam2.1	EG_81	Egypt	13_A2_84	266	272	0	205	213	0	154	156	160	166	170	273	279	0	192	192	134	136	154	341	341	173	173	284	294	0	240	244	0	260	266	0
EG_11_82_Tam21.1	EG_82	Egypt	23_A1_12	266	268	270	213	217	0	142	156	210	170	170	270	279	0	192	192	136	210	0	331	341	173	175	288	294	0	244	244	0	260	266	0
EG_11_83_Sha1.1	EG_83	Egypt	13_A2_84	266	272	0	205	213	0	154	156	160	166	170	273	279	0	192	192	134	136	154	341	341	173	173	284	294	0	240	244	0	260	266	0
EG_11_84_Tam22.1	EG_84	Egypt	13_A2_84	266	272	0	205	213	0	154	156	160	166	170	273	279	0	192	192	134	136	154	341	341	173	173	284	294	0	240	244	0	260	266	0
EG_11_86_TAM24.1	EG_86	Egypt	23_A1_12	266	268	270	213	217	0	142	160	210	170	170	270	279	0	192	192	136	210	0	331	341	173	175	288	294	0	244	244	0	260	266	0
EG_11_87_Men32.1	EG_87	Egypt	23_A1_15	266	268	270	213	217	0	156	210	0	170	170	270	279	0	192	192	136	214	0	331	341	173	175	288	294	0	244	244	0	260	266	0
EG_11_88_Tam25.1S.F	EG_88	Egypt	13_A2_84	266	272	0	205	213	0	154	156	160	166	170	273	279	0	192	192	134	136	154	341	341	173	173	284	294	0	240	244	0	260	266	0
EG_11_89_Sak1.1	EG_89	Egypt	23_A1_12	266	268	270	213	217	0	142	156	210	170	170	270	279	0	192	192	136	210	0	331	341	173	175	288	294	0	244	244	0	260	266	0
EG_11_90_MAH3.1	EG_90	Egypt	13_A2_84	266	272	0	205	213	0	154	156	160	166	170	273	279	0	192	192	134	136	154	341	341	173	173	284	294	0	240	244	0	260	266	0
EG_11_91_SAK2.1	EG_91	Egypt	13_A2_84	266	272	0	205	213	0	154	156	160	166	170	273	279	0	192	192	134	136	154	341	341	173	173	284	294	0	240	244	0	260	266	0
EG_12_92_MAH4.1	EG_92	Egypt	13_A2_84	266	272	0	205	213	0	154	156	160	166	170	273	279	0	192	192	134	136	154	341	341	173	173	284	294	0	240	244	0	260	266	0
EG_12_93_MAH5.1	EG_93	Egypt	13_A2_84	266	272	0	205	213	0	154	156	160	166	170	273	279	0	192	192	134	136	154	341	341	173	173	284	294	0	240	244	0	260	266	0
EG_12_94_MAH6.1sf	EG_94	Egypt	13_A2_84	266	272	0	205	213	0	154	156	160	166	170	273	279	0	192	192	134	136	154	341	341	173	173	284	294	0	240	244	0	260	266	0
EG_12_95_SAK3.1	EG_95	Egypt	13_A2_84	266	272	0	205	213	0	154	156	160	166	170	273	279	0	192	192	134	136	154	341	341	173	173	284	294	0	240	244	0	260	266	0

EG_12_96_MAH7.1	EG_96	Egypt	13_A2_84	266	272	0	205	213	0	154	156	160	166	170	273	279	0	192	192	134	136	154	341	341	173	173	284	294	0	240	244	0	260	266	0
FDale33d	C_1	UK	1_A1	266	268	0	213	217	0	142	162	0	166	170	270	279	0	192	192	136	136	0	341	355	173	175	284	294	0	244	244	0	260	266	0
PCL_10_8070B	C_2	UK	13_A2_87	266	272	0	205	213	0	154	156	160	166	170	273	279	0	192	192	136	154	0	341	341	173	173	284	294	0	240	244	0	260	266	0
